# Targeting Notch1 signaling pathway positively affects the sensitivity of osteosarcoma to cisplatin by regulating the expression and/or activity of Caspase family

**DOI:** 10.1186/1476-4598-13-139

**Published:** 2014-06-03

**Authors:** Lei Wang, Fangchun Jin, An Qin, Yongqiang Hao, Yufeng Dong, Shengfang Ge, Kerong Dai

**Affiliations:** 1 Department of Orthopaedics, Ninth People’s Hospital, Shanghai JiaoTong University School of Medicine, 639 Zhizaoju Road, Shanghai 200011, People’s Republic of China; 2 Department of Orthopaedics and Rehabilitation, Center for Musculoskeletal Research, University of Rochester School of Medicine and Dentistry, Rochester, NY 14642, USA; 3 Department of Ophthalmology, Ninth People’s Hospital, Shanghai JiaoTong University School of Medicine, 639 Zhizaoju Road, Shanghai 200011, People’s Republic of China

**Keywords:** Osteosarcoma, Notch1 signaling pathway, Targeting regulation, Cisplatin sensitivity, Caspase family

## Abstract

**Background:**

The introduction of cisplatin has improved the long-term survival rate in osteosarcoma patients. However, some patients are intrinsically resistant to cisplatin. This study reported that the activation of Notch1 is positively correlated with cisplatin sensitivity, evidenced by both clinical and in vitro data.

**Results:**

In this study, a total 8 osteosarcoma specimens were enrolled and divided into two groups according to their cancer chemotherapeutic drugs sensitivity examination results. The relationship between Notch1 expression and cisplatin sensitivity of osteosarcoma patients was detected by immunohistochemistry and semi-quantitative analysis. Subsequently, two typical osteosarcoma cell lines, Saos-2 and MG63, were selected to study the changes of cisplatin sensitivity by up-regulating (NICD1 plasmid transfeciton) or decreasing (gamma-secretase complex inhibitor DAPT) the activation state of Notch1 signaling pathway. Our results showed a significant correlation between the expression of Notch1 and cisplatin sensitivity in patient specimens. In vitro, Saos-2 with higher expression of Notch1 had significantly better cisplatin sensitivity than MG63 whose Notch1 level was relatively lower. By targeting regulation in vitro, the cisplatin sensitivity of Saos-2 and MG63 had significantly increased after the activation of Notch1 signaling pathway, and vice versa. Further mechanism investigation revealed that activation/inhibition of Notch1 sensitized/desensitized cisplatin-induced apoptosis, which probably depended on the changes in the activity of Caspase family, including Caspase 3, Caspase 8 and Caspase 9 in these cells.

**Conclusions:**

Our data clearly demonstrated that Notch1 is critical for cisplatin sensitivity in osteosarcoma. It can be used as a molecular marker and regulator for cisplatin sensitivity in osteosarcoma patients.

## Introduction

Osteosarcoma is the most common primary malignant bone tumor. It has a typical age related incidence rate, with a first peak occurring in the second decade (3.1-4.2 per 1,000,000 person years) and a second peak occurring in patients older than sixty (3.3-4.6 per 1,000,000 person years) [1-3]. Current treatments for osteosarcoma include combined approaches of surgery dissection and systemic chemotherapy. It is undeniable that the introduction of adjuvant and neoadjuvant chemotherapies has improved the long-term survival rate impressively [4-6]. For example, the use of cisplatin has been proved to be a useful chemotherapeutic method for preoperative induction therapy for osteosarcoma. Good responders to preoperative cisplatin chemotherapy showed a better survival rate [7]. Besides, it was also well summarized that the inclusion of cisplatin had better outcome for high grade osteosarcoma [8].

Cisplatin is an effective antitumor agent with a wide spectrum of activity against human solid tumors [9-13]. Generally, it can form bivalent adducts with nucleophilic sites on purines in DNA, yield predominantly DNA intra-strand cross links between adjacent purines [14], and thus exert antitumor effects. However, while some common tumors are sensitive to cisplatin treatment, others are instrinsically resistant to cisplatin [15], leading to failure in the curative therapy. However, the underlying mechanisms of cisplatin resistance are still unclear. Identifying molecules that contribute to cisplatin resistance would be necessary and significant for the optimization of curative effects.

The Notch signaling pathway is an evolutionarily conserved ligand-receptor signaling system that regulates cell proliferation, survival, apoptosis and differentiation [16,17]. Four Notch receptors have been identified in mammals, namely Notch1-4. Upon the binding of a ligand (jagged 1, jagged 2, delta-like 1 or delta-like 4) to the cell surface Notch receptors (Notch1-4), the Notch intracellular domain (NICD) will be cleaved by the gamma-secretase complex and translocated into the nucleus to induce the expression of downstream targeting genes including Hes1, Hes5, Hes7, Hey1, Hey2 and HeyL *etc.*[18]. Dysfunction of Notch signaling pathway may lead to anomalous differentiation or undifferentiation, ultimately causing these cells toward malignant transformation. Indeed, many observations suggested that alterations in Notch signaling are associated with many human cancers [19-23]. In addition, Notch signaling pathway is also involved in chemotherapy drug-resistance. For instance, Notch1 plays an important role in the mechanisms of cisplatin resistance in several malignant tumors, such as head and neck squamous cell tumors, colorectal tumors and ovarian cancer [24]. However, the roles of Notch1 signaling in osteosarcoma and chemotherapy response have not been elucidated yet. Therefore, this study aims to determine the roles of Notch1 signaling pathway in osteosarcoma, as well as the mechanisms of cisplatin response in osteosarcoma.

## Materials and methods

### Patients and specimens

Conventional osteosarcoma patients involved in this study were hospitalized in the Department of Orthopedic Surgery of the Ninth People’s Hospital of Shanghai, China between September 2010 and March 2012. All patients who enrolled provided informed consent in accordance with our institution’s regulatory requirements, and we conducted the study according to guidelines of the ethic committee of Ninth People's Hospital of Shanghai Jiaotong University.

In all cases, diagnosis of conventional osteosarcoma was established by clinical characteristics, radiological findings and pathological examination. Participants were randomly selected from our sample library, included seven patients (six males, one female) with median age of 31.6 years (range: 17–58 years). However, it is worthwhile to explain that one of these patients suffered a tumor recurrence after surgery operation within one year. Therefore, we have a total of eight histological specimens: sample 001–008 (sample 003 and sample 005 were both from the recurrence patient) which were all obtained after lesion excision. Then according to the inhibition rate of cisplatin in the tumor susceptibility test results, the histological specimens of these patients were divided into two groups: cisplatin sensitive group (cisplatin sensitivity 50%-90%) and cisplatin insensitive group (cisplatin sensitivity 0-30%). The two groups had no significant difference in composition regarding age or sex (P > 0. 05).

### Cell culture

Human osteosarcoma cell lines, Saos-2 and MG63 cells were purchased from the Shanghai Institute of Biochemistry and Cell Biology (Shanghai, China). They were authenticated using DNA fingerprinting (variable number of tandem repeats), confirming that no cross-contamination occurred during this study. The cell lines were used within 6 months of resuscitation. Cells were grown in α-MEM (GIBCO, Grand Island, NY, USA) supplemented with 10% FBS (Hy-clone, Tauranga, New Zealand) and antibiotics (penicillin 100 U/ml, streptomycin 100 lg/ml; Hyclone, Logan, UT,USA) in 37°C humidified atmosphere with 5% CO_2_.

### Immunohistochemistry and immunocytochemistry

Immunohistochemical staining for Notch1 and HES1 were performed on 5 μm thick formalin-fixed paraffin embedded sections. Deparafinization and rehydration were performed in xylene and ethanol solutions (reducing concentration 95%-70%). Sections were incubated in H_2_O_2_ solution (3% H_2_O_2_ in PBS buffer) for 30 minutes to block endogenous peroxydase. Antigen retrieval were performed in procedure with the retrieval buffer (PH = 9.0, TRIS 20 mmol/L, EDTA 0.05 mmol/L, 0.05% Tween 20) in 99°C bath for 20 minutes. Sections were then incubated with the primary antibody: anti-HES1 (diluted 1:250, Epitomics, USA) and anti-Notch1 (diluted 1:50, Epitomics, USA) at 4°C overnight. After rinsing with the PBS buffer, the secondary antibody (MaxVision TM HRP-Polymer anti-Rabbit IHC Kit, Maixin.Bio, China) was applied for 15 minutes at room temperature (RT). DAB (Maixin.Bio, China) solution was used for chromogen. At last, the Sections were counterstained with Hematoxylin (Sigma) to identify nuclei. Immunohistochemistry with the secondary antibody alone without primary antibody was carried out as a control. The images were acquired using a microscope (Leica DM 4000B) with BioQuant OSTEO II software (BioQuant Image AnalysisCorporation, Nashville, TN).

Immunocytochemistry for Notch1 of Saos-2 and MG63 cells were performed in 6-well plate. Supernatant was discarded and the plate was washed by PBS buffer for three times. Then 0.2% Triton X-100 was added to penetrate cytomembrane for 5 minutes and 5% BSA was used for 30 minutes to block unspecific binding of the antibodies. Subsequently, sections were incubated with the primary antibody against Notch1 (diluted 1:100, Epitomics, USA) at 4°C overnight. After washing three times with PBS buffer, the cells were incubated with anti-rabbit Alexa594 (1:500, Molecular Probes, USA) for 30 minutes. DAPI (300 nM, Molecular Probes, USA) was used to counterstain the nuclei. At last, the images were acquired using an inverted fluorescence microscope (IX71, Olympus, Japan) with DP Controller software.

### Activation and inhibition of Notch1 signaling pathway of Saos-2 and MG63 cells

For the activation of Notch1 signaling pathway of Saos-2 and MG63 cells, the Notch1 intracellular domain (NICD-1) plasmid which was cloned 2.4 kb NICD-1 fragment to pcDNA3.1 vector using forward primer: 5′-CACC ATG GTG CTG CTG TCC CGC AA GCGCC and reverse primer: 5′- TGC TTT AAA TGC CAC AGG AAT GTG GG was used to transfect into the two cell lines according to the protocols of Lipofectamine™ Reagent (Invitrogen, USA). Three days later, we detected the expression of HES1 mRNA, the main downstream aim target of Notch signaling pathway, as the indication of the activation of Notch1 signaling pathway by the plasmid. Subsequently, these cells were seeded into 96-well plate for the follow-up studies.

LY-374973, N-[N-(3,5-Difluorophenacetyl)-L-alanyl]-S-phenylglycine t-butyl ester (DAPT) (ENZO Life Sciences, USA), the inhibitor of γ-secretase complex in Notch signaling pathway, was used to non-specific inhibit the Notch1 signaling pathway of Saos-2 and MG63 cells. The cells were incubated in DAPT (100 μmol/L) in the 6-well/96-well plates for 24 hours. And then DAPT was removed and the cells were washed with fresh medium twice before cisplatin added.

### Real-time PCR

Total RNA was isolated by using the AxyPrepTM Multisource Total RNA Miniprepkit (Axygen, USA). An equivalent amount of RNA was converted into complementary DNA (cDNA) with PrimeScript™ RT reagent Kit (Takara,Japan). Subsequently, Real-time PCR was performed using an ABI 7500 Sequencing Detection System and SYBR®Premix Ex Taq (Takara, Japan). All of the procedures were performed according to the manufacturer’s protocols. Cycling condition was as follows: 40 cycles at 95°C for 5 seconds and 60°C for 34 seconds. The primer sequences are, for human Notch1: forward 5′-GGA GGC ATC CTA CCC TTT TC-3′, and reverse 5′-TGT GTT GCT GGA GCA TCT TC-3′; for human HES1: forward 5′-CTC TCT TCC CTC CGG ACT CT-3′ and reverse 5′-AGG CGC AAT CCA ATA TGA AC-3′; for human Caspase3: forward 5′-TTT TTC AGA GGG GAT CGT TG-3′ and reverse 5′-CGG CCT CCA CTG GTA TTT TA-3′; for human Caspase8: forward 5′-TGC AGG GTC TCA CTC TGT TG-3′ and reverse 5′-CAA AAA TCA GCC ATG TGT GG-3′; for human Caspase9: forward 5′-CTA GTT TGC CCA CAC CCA GT-3′; and for human GAPDH: forward 5′-CCT GCA CCA CCA ACT GCT TA-3′ and reverse 5′-AGG CCA TGC CAG TGA GCT T-3′. The comparative 2-ΔΔCT method was used to calculate the relative expression level of each target gene with GAPDH as the housekeeping gene.

### Western blot

At the end of incubation, cells were washed with PBS and dissolved in lysis buffer containing protease inhibitor. Equal amount of proteins in cell homogenates were subjected to 10% SDS–PAGE and transferred to 0.22 μm PVDF membranes. The membranes were blocked with 5% free fat milk at room temperature for 1 h and then incubated with primary antibodies (all in accordance with 1:1,000 dilution), including rabbit monoclonal anti-Notch1 intracellular domain, rabbit monoclonal anti- Pro-Caspase3/cleaved Caspase3, rabbit monoclonal anti-Caspase8 and mouse monoclonal Pro-Caspase9/cleaved Caspase9 (all were purchased from Cell Signaling Technology, USA), overnight at 4°C, after 3 washes in TBST, the membranes were incubated with anti-rabbit or anti-mouse IgG for 1 h at room temperature. After washing, the membranes were incubated with enhanced chemiluminescence system (ECL) detection kit (Amersham Life Science, Little Chalfont, UK). Positive immunoreactive bands were quantified densitometrically, normalized by GAPDH.

### Remaining cells test after treated with cisplatin by CCK-8 assay

A cell count kit-8 (CCK-8, Dojindo, Japan) was employed in this experiment to quantitatively evaluate the remaining cells viability after Saos-2 and MG63 cells whose Notch1 signaling pathway was activated or inhibited were treated with cisplatin (10 μg/ml, 5 μg/ml and 2.5 μg/ml). Cisplatin application concentration was calculated according to the clinical treatment program (20 mg/m^2^ ≈ 5 μg/ml). Approximately 7 × 10^3^ cells were seeded on each film placed in the 96-well plates. The culture medium was removed and the cells were washed with PBS before CCK-8 examination. Subsequently, 100 μl α-MEM medium and 10 μl CCK-8 solution were added to each sample, followed by incubation at 37°C for 2.5 hours. The optical density (OD) at 450 nm was determined using a microplate reader (BIO-TEK, USA). At last, all the CCK8 results in our study were divided by their self-control group to obtain the ratio of remaining cell and the proliferation rate: $\mathrm{Ratio}\;\mathrm{of}\;\mathrm{remaining}\;\mathrm{cell}\;\left(\mathrm{relative}\;\mathrm{sensitivity}\;\mathrm{to}\;\mathrm{cisplatin}\right)=\frac{\mathrm{the}\;24h/36h\;\mathrm{absorption}\;\mathrm{value}\;\mathrm{of}\;\mathrm{cisplatin}\;\mathrm{treatment}\;\mathrm{group}}{\mathrm{the}\;24h/36h\;\mathrm{absorption}\;\mathrm{value}\;\mathrm{of}\;\mathrm{cisplatin}\;\mathrm{untreatment}\;\mathrm{control}\;\mathrm{group}}$; $\mathrm{Proliferation}\;\mathrm{rate}=\frac{\mathrm{the}\;24/36h\;\mathrm{absorption}\;\mathrm{value}\;\mathrm{of}\;\mathrm{cisplatin}\;\mathrm{untreatment}\;\mathrm{group}}{\mathrm{the}\;0h\;\mathrm{absorption}\;\mathrm{value}\;\mathrm{of}\;\mathrm{cisplatin}\;\mathrm{untreatment}\;\mathrm{group}}.$

### Analysis of apoptosis

Cells were seeded at 200,000 cells per well in six-well plates. NICD-1 plasmid transfection cells were harvested at 24 hours after 5 μg/ml cisplatin treatment and 36 hours for DAPT treatment cells. Cells were washed twice with cold PBS and then re-centrifuged the washed cells, discarded the supernatants and resuspended the cells in 1X annexin-binding buffer. Early apoptosis was detected by staining with Alexa Fluor® 488 annexin V and Propidium iodide labeling using the Vybrant® Apoptosis Assay Kit #2 (Invitrogen, USA). FACS was performed using a FACScan flowcytometer (Beckman-Altra, USA). Data were acquired using CELL Quest software.

### Statistics analysis

Each sample was analysed in triplicate, and experiments were repeated three times. Mean, standard deviation (SD), and P values base on the 2-tailed t test were calculated with Excel X (Microsoft). Differences were considered significant at P < 0.05.

## Results

### Osteosarcoma patients with higher Notch1 expression are more sensitive to cisplatin treatment

**Figure 1:**
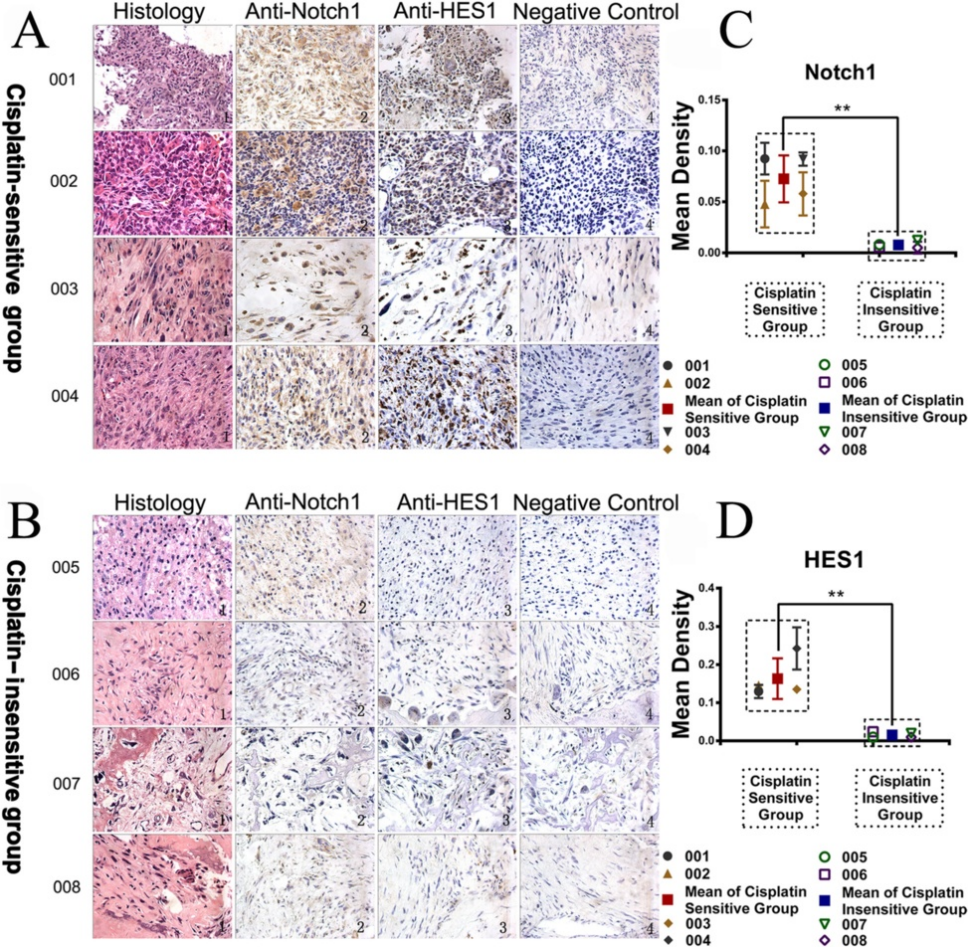
**Immunohistochemistry showed the expression of Notch1 and HES1 in cisplatin sensitive group and insensitive group.** The histopathology revealed the irregular nuclei, some spindle cells, and even destroyed bone (**A** and **B**, Column 1). Immunohistochemical examination of Notch1 and HES1 were carried out in different patients. There is significant more expression of Notch1 and HES1 in cisplatin sensitive patients than cisplatin insensitive patients in gross appearance (**A** and **B**, Column 2 and 3) and semi-quantitative scatter plot below **(C and D)**. Column 4 in **A** and **B** was the negative control with the secondary antibody alone without primary antibody. (**P < 0.01).

Osteosarcoma cells from different patients exhibited different response to cisplatin treatment. According to the inhibition rate of cisplatin and carboplatin on tumor cells from different patients, all the osteosarcoma patients were divided into two groups: cisplatin sensitive group (samples 001–004) and cisplatin insensitive group (samples 005–008). Approximately 63.88 ± 14.57% of the osteosacoma cells respond to cisplatin treatment in the sensitive group while 6.88 ± 6.33% respond to cisplatin treatment in the insensitive group (Additional file 1: Table S1). Then, the expression profile of Notch1 and HES1 were analyzed by immunohistochemical staining (Figure 1A and B). As shown in Figure 1, intense immunoreactivity of Notch1 and HES1 was observed in cisplatin sensitive patients (samples 001–004). On the contrary, low expression of Notch1 and HES1 was observed in specimens from cisplatin insensitive patients (samples 005–008). It was worth noting that sample 003 and sample 005 were from the same patient at different stage. Sample 003 was obtained from primary osteosarcoma while sample 005 was taken from recurrent osteosarcoma. Interestingly, sample from the primary osteosarcoma were sensitive to cisplatin treatment (inhibition rate: 54.28%) with higher Notch1 and HES1 expression. In contrast, sample from the recurrent osteosarcoma were insensitive (inhibition rate: 0.00%) to cisplatin treatment with low Notch1 and HES1 expression. Together, these data suggested that cisplatin sensitivity was positively correlated with Notch1 expression.

### Osteosarcoma cell line with higher Notch1 expression is more sensitive to cisplatin treatment

**Figure 2:**
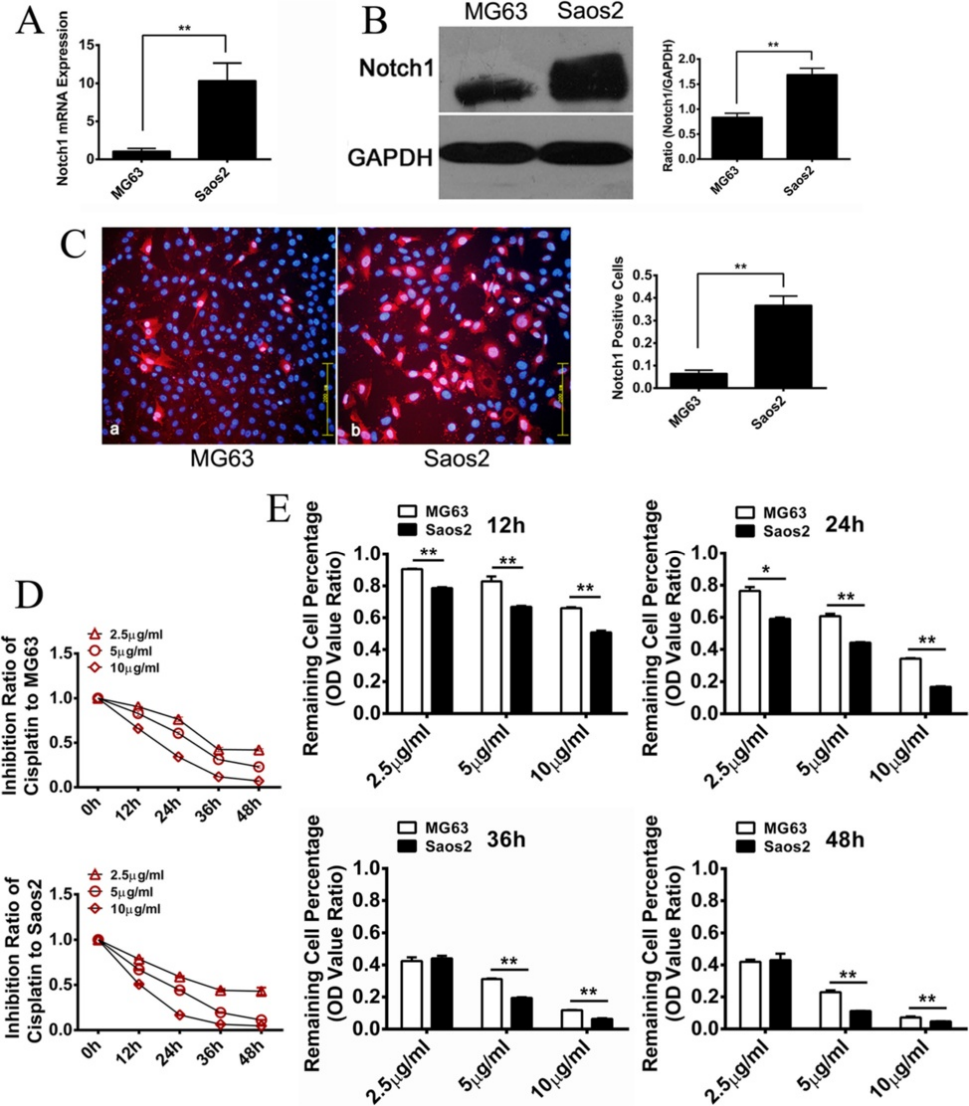
**The expression of Notch1 in MG63 and Saos-2 cell lines and their sensitivity to cisplatin.** Real-time PCR revealed that Saos-2 cells had approximately 7 fold higher Notch1 expression than MG63 cells **(A)**. We further carried out Western Blot and immunocytochemistry to determine the protein expression in MG63 and Saos-2. The level of Notch1 in Saos-2 is significantly higher than MG63 (error bar means s.d.) **(B and C)**. Both two cell lines showed dose and time dependent response to cisplatin **(D)**. Saos-2 performed significantly higher sensitivity to cisplatin **(E)**. (*P < 0.05, **P < 0.01).

In order to verify the positive relationship between the expression of Notch1 and cisplatin sensitivity in osteosarcoma, two osteosarcoma cell lines, Saos-2 and MG63, were analyzed *in vitro*. Both two cell lines are the most commonly used osteosarcoma cell lines. They have substantially similar cell morphology, cell cycle and culture conditions, which will make it easy for us to unify the cisplatin dose and time points in subsequent experiments. Initial realtime PCR revealed Notch1 expression was approximately 7 fold higher in Saos-2 cells than MG63 cells (Figure 2A). This result was further confirmed by Western Blot and immunocytochemistry, showing significantly higher Notch1 expression in Saos-2 cells (Figure 2B and C). Both Saos-2 and MG63 cell lines showed a dose and time dependent response to cisplatin treatment (10 μg/ml, 5 μg/ml, and 2.5 μg/ml) after 12 h, 24 h, 36 h and 48 h (Figure 2D). Inteterstingly, Saos-2 cells were more sensitive to cisplatin treatment than MG63 cells, as evidenced by significant lower cell viability in the Saos-2 group after cisplatin treatment (Figure 2E). Collectively, these data indicated that Saos-2 with higher Notch1 expression was more sensitive to cisplatin treatment while MG63 with lower Notch1 expression was relatively insensitive to cisplatin treatment.

### Activation of Notch1 signaling pathway sensitizes osteosarcoma cell lines to cisplatin treatment

**Figure 3:**
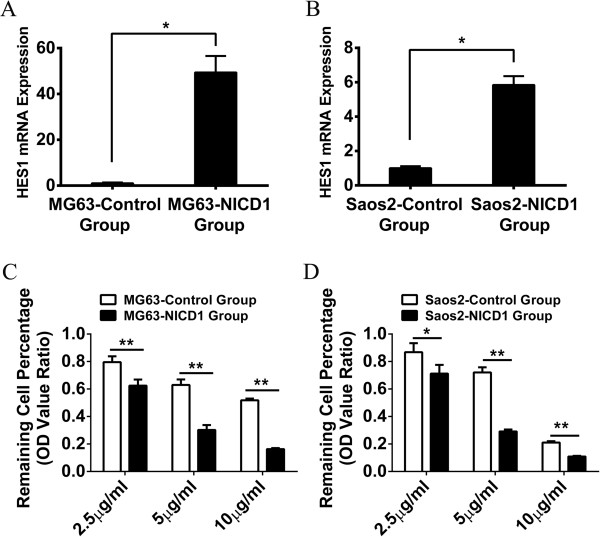
**Activation of Notch1 signaling pathway increased the sensitivity of MG63 and Saos-2 to cisplatin.** Real-time PCR indicated that HES1 mRNA in MG63 and Saos-2 had significantly more expression after NICD-1 plasmid transfection **(A and B)**. The sensitivity of MG63 and Saos-2 to cisplatin has significantly increased after the activation of Notch1 signaling pathway **(C and D)**. (*P < 0.05; **P < 0.01).

The activation of notch intracellular domain (NICD) is a key step for Notch signaling pathway. Thus, the NICD-1 plasmid was transfected into MG63 or Saos-2 cells to activate their Notch1 signaling pathway. The activation of Notch1 signaling was confirmed by the expression of targeting gene HES1 which increased approximately 50-fold and 5.8-fold in MG63 and Saos-2 cells respectively (Figure 3A and B). Subsequently, the activated cell lines were treated with different concentrations of cisplatin (10 μg/ml, 5 μg/ml and 2.5 μg/ml) for 24 hours. Interestingly, both activated MG63 and Saos-2 exhibited significantly increased sensitivity to cisplatin compare to their control groups (Figure 3C and D). More than 80% of the activated MG63 cells (MG63-NICD-1) respond to cisplatin at 10 μg/ml while only about 50% of the MG63 cells are sensitive to cisplatin treatment at the same dose. Similar difference was observed in the Saos-2 cell lines. Additionally, in order to exclude the influence of the plasmid on cell proliferation, the 24 h proliferation rate was also examined in the two cell lines with pure plasmid transfection. There was no significantly difference between the NICD-1 transfected group and control group in MG63. Saos-2 with NICD-1 transfection showed a higher proliferation rate (Additional file 2: Figure S1). Together, these results suggested that the activation of Notch1 signaling pathway sensitized osteosarcoma cells to cisplatin treatment.

### Inhibition of Notch1 signaling pathway desensitizes osteosarcoma cell lines to cisplatin treatment

**Figure 4:**
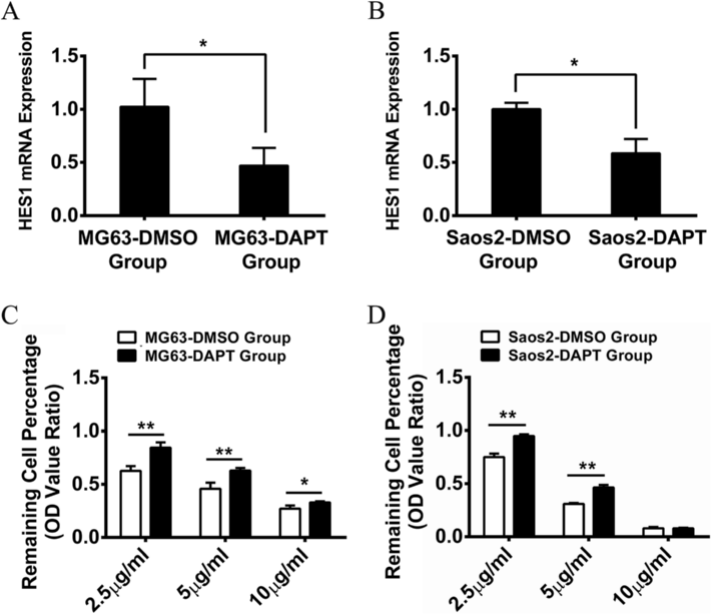
**Inhibition of Notch1 signaling pathway decreased the sensitivity of MG63 and Saos-2 to cisplatin.** Real-time PCR indicated that HES1 mRNA in MG63 and Saos-2 had significantly less expression after DAPT treatment **(A and B)**. The sensitivity of MG63 and Saos-2 to cisplatin has significantly decreased after the inhibition of Notch1 signaling pathway **(C and D)**. (*P < 0.05; **P < 0.01).

In order to further confirm our previous results, DAPT, the inhibitor of γ-secretase complex in Notch signaling pathway, was used to inhibit Notch1 signaling pathway. After treatment with DAPT for 24 hours, the expression of HES1 mRNA was measured by Real-time PCR to validate the inhibition efficiency of DAPT. As shown in Figure 4, HES1 was suppressed approximately 50% in MG63 and 40% in Saos-2 after the inhibition of Notch1 signaling pathway (Figure 4A and B). Different concentrations of cisplatin (10 μg/ml, 5 μg/ml and 2.5 μg/ml) were used to treat the inhibited MG63 and Saos-2 cells. Consistent with previous findings, we found that there were more alive cells in the DAPT inhibition group than the control group, indicating that MG63 and Saos-2 became relatively insensitive to cisplatin after the inhibition of Notch1 signaling pathway (Figure 4C and D). Additionally, in order to exclude the influence of DAPT on the results above, the proliferation rates were also examined in the two cell lines with DAPT treatment only. The result revealed that two cell lines showed no significant change in proliferation in the follow-up 36 hours after DAPT being removed (Additional file 3: Figure S2). Taken together, we demonstrated that suppression of Notch1 signaling pathway desensitized osteosarcoma cells to cisplatin treatment.

### After targeted regulation of Notch1 signaling pathway, osteosarcoma cells apoptosis changes under the cisplatin effect

**Figure 5:**
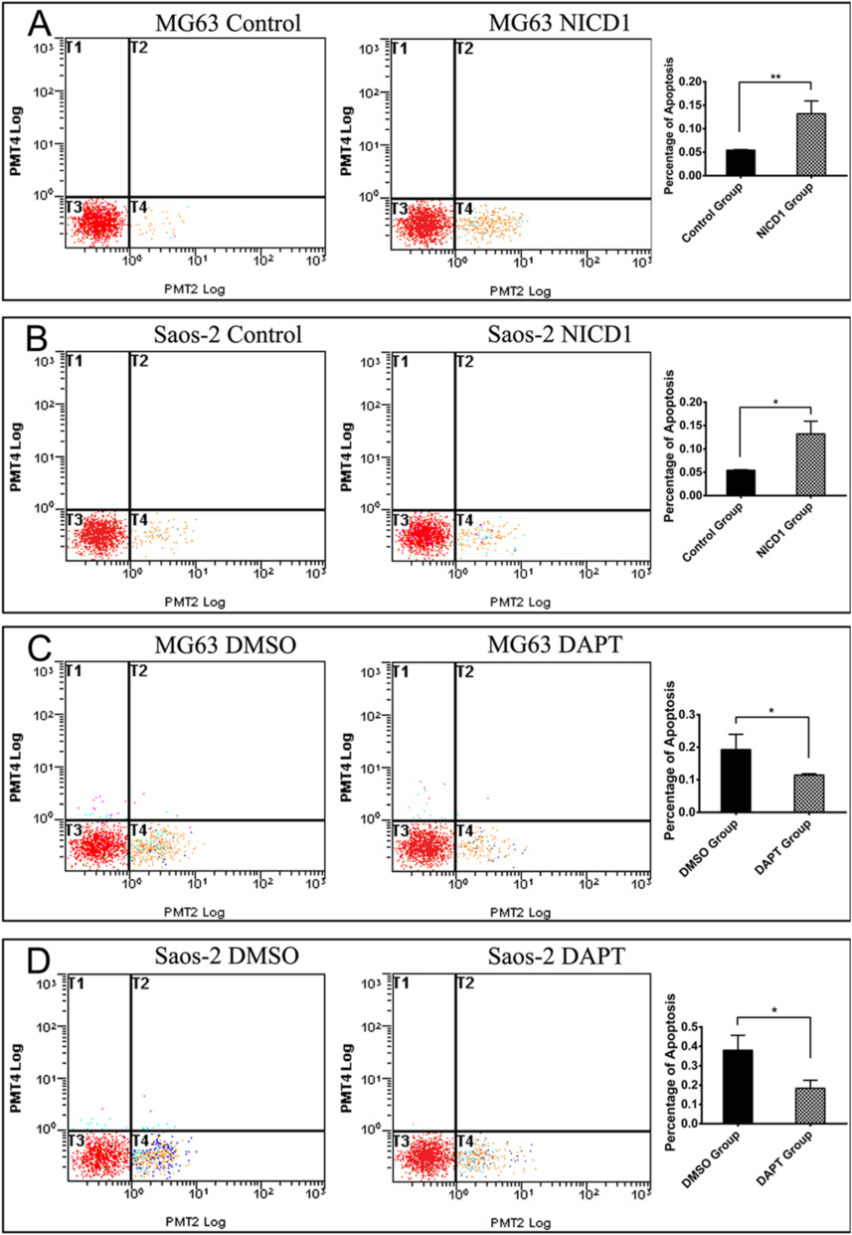
**Notch1 signaling pathway regulated cisplatin sensitivity of MG63 and Saos-2 via changing the apoptosis effect.** After overexpression of NICD-1, cisplatin could induce more apoptosis in MG63 and Saos2 cells than their control group **(A and B)**. When Notch1 signaling pathway was inhibited by DAPT, cisplatin induced fewer apoptosis cells in both osteosarcoma cell lines **(C and D)**. (*P < 0.05; **P < 0.01).

To better understand the effect of Notch1 signaling pathway on cisplatin-sensitivity in osteosarcoma, we examined the apoptosis rate induced by cisplatin. Our flow cytometry results showed that cisplatin induced approximately more than one fold apoptosis in NICD-1 plasmid activated cells (MG63 13.21% and Saos-2 8.09%) than control cells (MG63 5.45% and Saos-2 3.99%) after 24 hour cisplatin treatment (Figure 5A and B). In contrast, cisplatin induced apoptosis in 18.37% of DAPT inhibited MG63 cells and 11.40% of DAPT inhibited Saos-2 cells, which was much less than their control group (37.97% of MG63 and 19.26% of Saos-2) (Figure 5C and D). Collectively, these results indicated that Notch1 activation might enhance cisplatin induced osteosarcoma cell apoptosis.

### Inhibition of Notch1 signaling pathway decreases the expression and/or activity of Caspase3, Caspase8 and Caspase9 in osteosarcoma cells even under the effect of cisplatin

**Figure 6:**
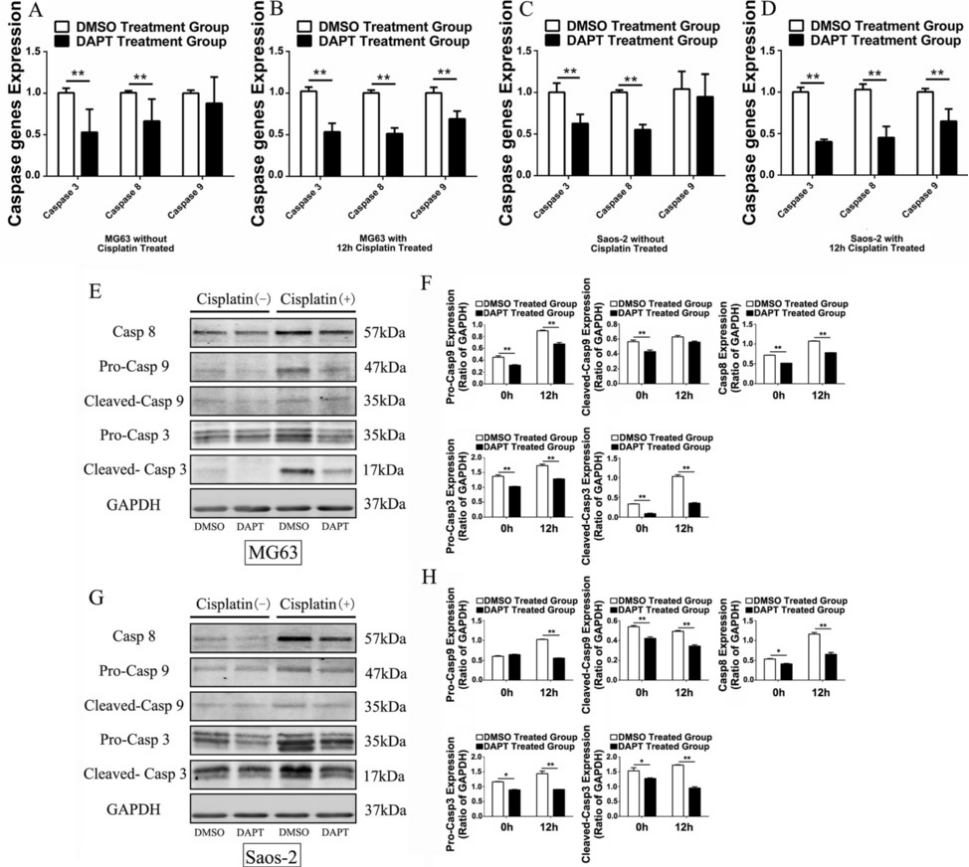
**Inhibition of Notch1 signaling pathway decreased the expression and/or activity of Caspase3, Caspase8 and Caspase9.** In the two cell lines, realtime PCR indicated that the gene expression of Caspase3, Caspase8 and Caspase9 were reduced to some extent after a simple DAPT treatment for 24 hours. Further reduction was observed with cisplatin subsequent treatment for 12 hours **(A, B, C and D)**. Simultaneously, western blot was carried out to detect the protein expression of Pro-Casp3, Cleaved-Casp3, Casp8, Pro-Casp9 and Cleaved-Casp9. The expression of these several proteins significantly decreased after DAPT treatment in MG63 and Saos-2 cells. Further, such difference between two groups could be obviously magnified after cisplatin treatment **(E, F, G and H)**. (*P < 0.05; **P < 0.01).

Cell apoptosis is a complex process that involves a variety of regulatory mechanisms. However, it is generally appreciated that Caspase family plays a crucial role in the regulation of cell apoptosis. Therefore, the expression and activity of Caspase3, one of the most important apoptosis executioners, Caspase8 and Caspase9, the most critical initiators of Caspase3, were detected in this part. Initially, realtime PCR revealed that Caspase3, Caspase8 and Caspase9 gene expression displayed different degrees of reduction with a simple DAPT treatment for 24 hours in MG63 and Saos-2 cells (Figure 6A and C). However, the expression of each Caspase gene was further reduced rather than reversed in DAPT group compared to DMSO group after a subsequent treatment with cisplatin for 12 hours (Figure 6B and D). Then, we simultaneously detected the protein expression of Caspase3 (Pro-Casp3), active Caspase3 (Cleaved-Casp3), Caspase8 (Casp8), Caspase9 (Pro-Casp9) and active Caspase9 (Cleaved-Casp9) in MG63 and Saos-2. Changes in the expression of Caspase3, Caspase8 and Caspase9 were broadly consistent with the genes, but they were still a little different. After treatment with DAPT for 24 hours with or without cisplatin reprocessing, the protein expressions of Pro-Casp3 and Cleaved-Casp3 which were likely to be the critical molecules affected by Notch1 signaling pathway were both significantly lower than the control group in MG63 and Saos-2 cells (Figure 6E-H). However, although the expression of the other indicators including Casp8, Pro-Casp9 and Cleaved-Casp9 didn’t show an obvious difference observed by naked eye between the two groups when the cells were only treated with DMSO or DAPT, a statistically difference that the expression levels of these proteins in DAPT group were significantly lower than it in DMSO group did exist. And then such slight difference magnified after the cisplatin treatment for 12 hours (Figure 6E-H). Therefore, we concluded that inhibition of Notch1 signaling pathway even with cisplatin treatment in osteosarcoma cells might directly or indirectly decrease the expression and/or activity of Caspase family proteins, generally leading to reduced sensitivity to cisplatin at last.

## Discussion

The use of multiagent, intensive chemotherapy has proved to be a major development in osteosarcoma treatment. It has dramatically changed osteosarcoma from a malignancy with a modest survival rate to one in which at least two thirds of patients could be cured [25,26]. Cisplatin is one of the most common first-line chemotherapy drugs for osteosarcoma because of its DNA cross-linking activity [27]. However, not all osteosarcoma patients are sensitive to cisplatin treatment [28]. Therefore, correctly distinguishing patient response to cisplatin treatment would allow sensitive patients to benefit from chemotherapy and avoid non-responsive patients suffering from unnecessary side effects.

In our study, we for the first time identified that the expression of Notch1 and HES1 in osteosarcoma specimens were positively correlated with cisplatin sensitivity in osteosarcoma patients. *In vitro* study confirmed this finding that osteosarcoma cell line Saos-2 with higher Notch1 expression was more sensitive to cisplatin than MG63 with lower Notch1 expression. Subsequently, we consolidated such discovery by activating or suppressing Notch1 signaling pathway further. Activation of Notch1 signaling pathway significantly sensitized osteosarcoma cells to cisplatin treatment while inhibition of Notch1 signaling pathway desensitized these cells to cisplatin. We further revealed that changing the activity of Notch1 signaling pathway was able to affect the cell apoptosis to regulate the sensitivity of osteosarcoma to cisplatin, which might be mainly caused by the expression and/or activity changes of Caspase family proteins. As was shown in Figure 6, inhibition of Notch1 signaling pathway could significantly decrease the amount of Caspase family proteins, especially Pro-Caspase 3 and Cleaved-Caspase 3, after cisplatin treatment. Therefore, on the view of macroscopic, osteosarcoma cells showed decreased apoptosis and insensitivity to cisplatin. On the contrary, we speculated that activation of Notch1 signaling pathway would have the opposite effect on the expression and/or activity of Caspase family proteins, which could lead osteosarcoma more sensitive to cisplatin. Overall, our study comprehensively revealed that the expression of Notch1 was positively correlated with cisplatin sensitivity in osteosarcoma. Thus, Notch1 could be an effective molecular marker for predicting and even regulating the cisplatin sensitivity in osteosarcoma patients.

The highly conserved Notch signaling pathway plays important roles in controlling a wide variety of cell fate decisions and governs numerous developmental processes which affect the development and function of many organs. Notch signaling pathway has a consanguineous relationship with osteosarcoma in its pathogenesis, development, invasion, and metastasis in recent studies [23,29,30]. However, the influence of Notch signaling pathway on osteosarcoma chemotherapy sensitivity has hardly been reported before. Previous studies have reported activation of Notch1 signaling pathway was negatively related to cisplatin sensitivity in malignancies of head and neck squamous cell tumors [31,32]. Opposite to these findings, we reported that activation of Notch signaling pathway was positively related with cisplatin sensitivity in osteosarcoma. Notch can have either an oncogenic [19-23] or a tumour-suppressor [33,34] role. Thus, this might be epitomized by its different contributions to cancers in different tumor types. Therefore, we consider that the opposite findings from our study and previous studies may due to the different effects of Notch1 in different tumors. Osteosarcoma is a kind of malignancy derived from mesenchymal tissue. Therefore, we might also be able to comprehend the above results by drawing lessons from the effect of Notch signaling pathway in bone marrow mesenchymal stem cells (MSCs). Some researches showed that Notch signaling pathway undertook an important regulation action in the proliferation and differentiation of MSCs: activation of Notch signaling pathway could obviously inhibit the process of osteogenic and chondrogenic differentiation [35-38]. Thus, Notch signaling pathway would keep them in an undifferentiated or poorly differentiated state. Similarly, we would speculate that the activation of Notch1 signaling pathway may retain osteosarcoma cells in an undifferentiated stage, in which cell division and DNA replication process may be more frequent. When osteosarcoma cells are treated with chemotherapy drugs whose target is DNA, DNA damage and replication errors would be more likely to occur in this case, directly leading to the initiation of cell apoptosis. Indeed, poorly differentiated osteosarcoma cells tend to be more sensitive to chemotherapy in clinical. On the contrary, well-differentiated tumors are always insensitive to chemotherapy, which might be also the deeper reason for the phenomenon observed in our present study.

Cisplatin, one of the most common anti-neoplastic drugs, is always able to bring better prognosis for sensitive patients. Our study proved that Notch1 signaling pathway whose activity changes might directly or indirectly affect the expression and activity of Caspase family proteins was also a possible key regulator for cell apoptosis induced by cisplatin in osteosarcoma. That is to say, Notch1 signaling pathway possibly plays a vital role in the anti-neoplastic effect of drugs with potential apoptosis-inducing ability. However, the following points are still to be resovled about our research: 1) more patients samples should be collected to further verify the relationship between Notch1 signaling pathway and cisplatin sensitivity; 2) the intermediate molecular mechanisms on how Notch1 changing the expression and activity of Caspase family proteins should be better clarified; 3) more chemotherapy drugs with potential apoptosis-inducing ability should be involved to try to reveal the universal law.

## Conclusions

Our findings showed that Notch1 signaling pathway had a positive correlation with cisplatin sensitivity in osteosarcoma. This novel finding adds to the understanding of osteosarcoma chemotherapy sensitivity. Notch1 signaling pathway can be used as a molecular marker for cisplatin sensitivity in osteosarcoma patients.

## Competing interests

The authors declare that they have no competing interests.

## Authors’ contributions

LW, FJ, AQ and YD conducted the experiments and were involved in data analysis. LW helped with drafting the manuscript. YH designed the study, analyzed, and interpreted data, and drafted the manuscript. KD and SG revised the manuscript for intellectual content. All authors read and approved the final manuscript.

## Supplementary Material

Additional file 1: Table S1 The Inhibition Rate of Cisplatin on Osteosarcoma. The inhibition rate of cisplatin in osteosarcoma was measured. Based on these data, the samples were divided into two groups: cisplatin sensitive group (samples 001–004) and cisplatin insensitive group (samples 005–008). Click here for file

Additional file 2: Figure S1 The pure NICD1 plasmid transfection without cisplatin showed no obvious influence on the proliferation of MG63. On the contrary, it could significantly promote the proliferation of Saos-2. Click here for file

Additional file 3: Figure S2 Cells were only treated with DAPT and without cisplatin showed no obvious influence on the proliferation of MG63 and Saos-2. Click here for file
