# Supplementary material for: Targeting Notch1 signaling pathway positively affects the sensitivity of osteosarcoma to cisplatin by regulating the expression and/or activity of Caspase family
Source: Mol Cancer. 2014 Jun 3;13:139. doi: 10.1186/1476-4598-13-139 (PMC4110525; doi:10.1186/1476-4598-13-139)
Supplement: Additional file 1: Table S1 — The Inhibition Rate of Cisplatin on Osteosarcoma. The inhibition rate of cisplatin in osteosarcoma was measured. Based on these data, the samples were divided into two groups: cisplatin sensitive group (samples 001–004) and cisplatin insensitive group (samples 005–008). [file 1476-4598-13-139-S1.doc]

**Supplementary Table 1**

**The Inhibition Rate of Cisplatin on Osteosarcoma**

| **Patients** | |  | | **Cisplatin** |
| --- | --- | --- | --- | --- |
| **Sensitive patients** | **001** | | 57.16% | |
|  | **002** | | 58.50% | |
|  | **003** | | 54.28% | |
|  | **004** | | 85.58% | |
| **Insensitive patients** | **005** | | 3.02% | |
|  | **006** | | 11.88% | |
|  | **007** | | 12.61% | |
|  | **008** | | 0.00% | |

The inhibition rate of cisplatin in osteosarcoma was measured. Based on these data, the samples were divided into two groups: cisplatin sensitive group (samples 001-004) and cisplatin insensitive group (samples 005-008).
