# Supplementary figures and images for: Targeting Notch1 signaling pathway positively affects the sensitivity of osteosarcoma to cisplatin by regulating the expression and/or activity of Caspase family
Source: Mol Cancer. 2014 Jun 3;13:139. doi: 10.1186/1476-4598-13-139 (PMC4110525; doi:10.1186/1476-4598-13-139)

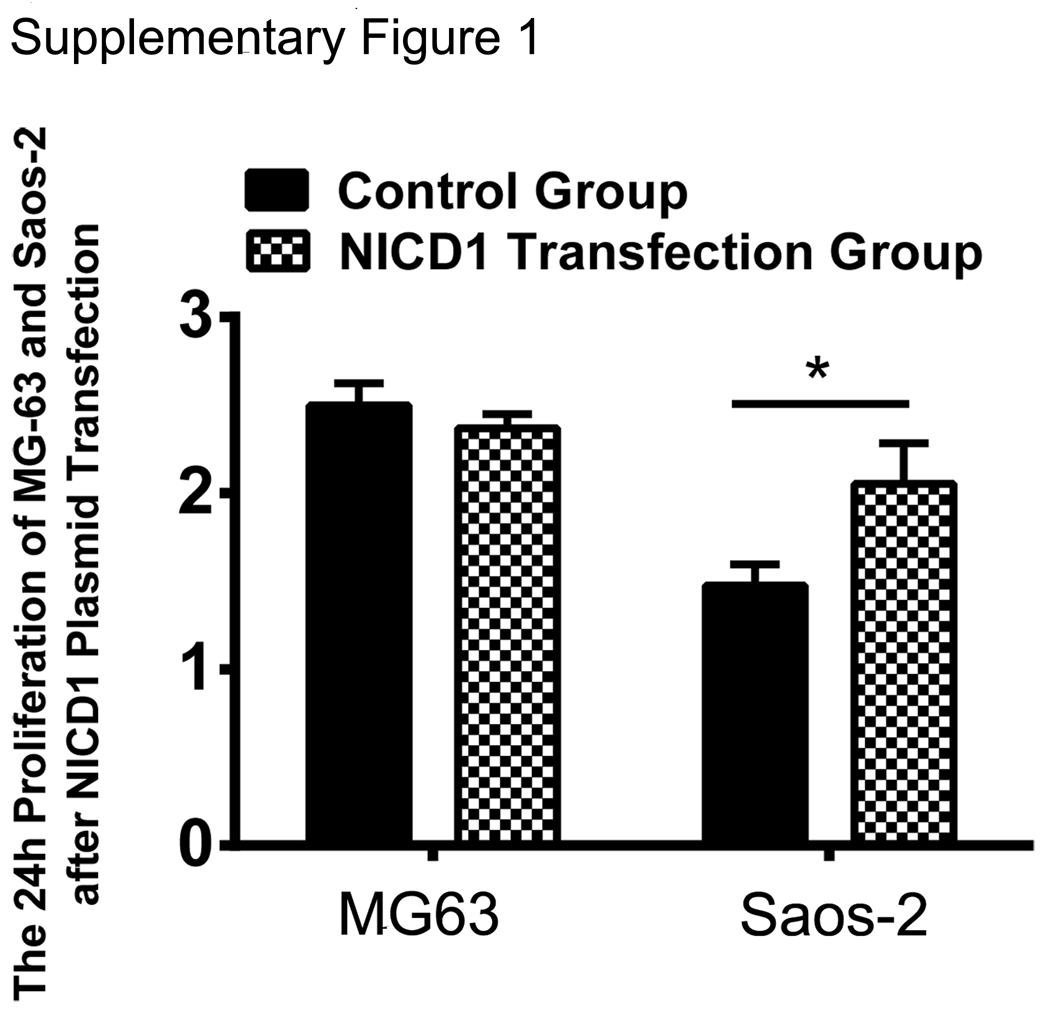

Supplement: Additional file 2: Figure S1 — The pure NICD1 plasmid transfection without cisplatin showed no obvious influence on the proliferation of MG63. On the contrary, it could significantly promote the proliferation of Saos-2. [file 1476-4598-13-139-S2.tiff]

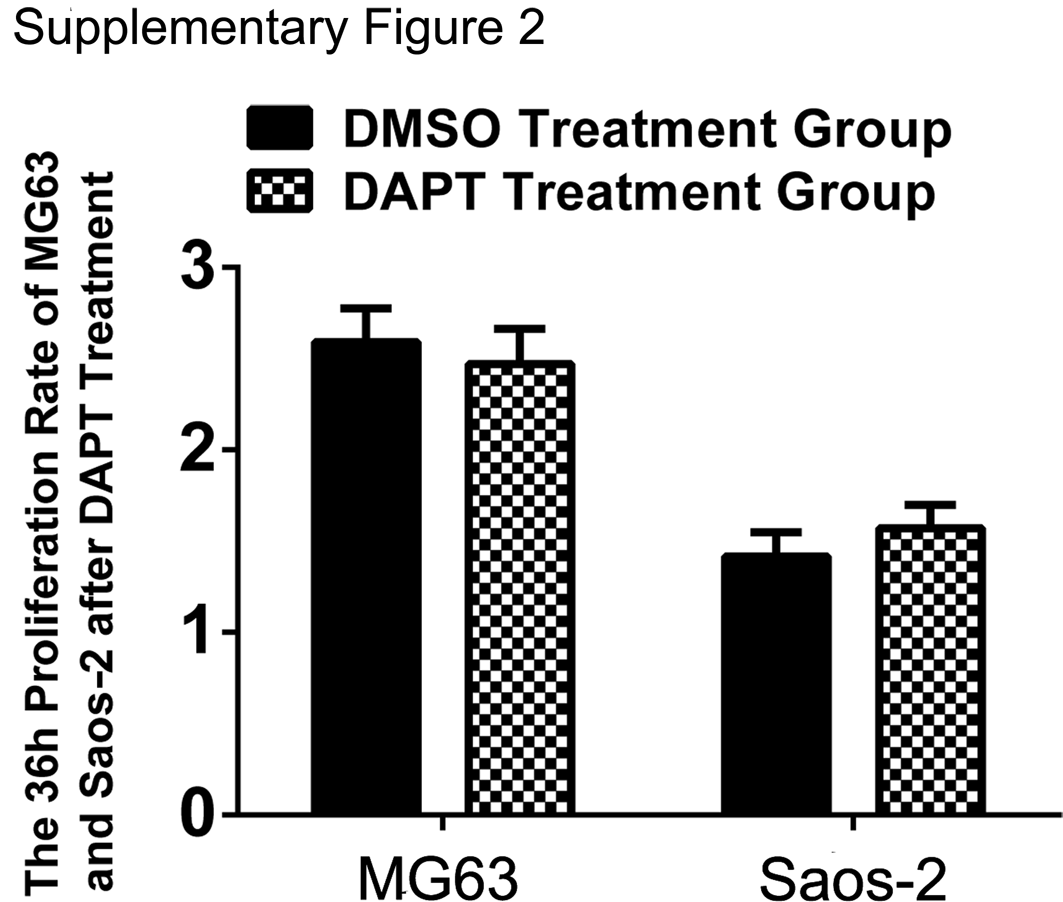

Supplement: Additional file 3: Figure S2 — Cells were only treated with DAPT and without cisplatin showed no obvious influence on the proliferation of MG63 and Saos-2. [file 1476-4598-13-139-S3.tiff]
